# Supplementary figures and images for: PD173074 blocks G1/S transition via CUL3-mediated ubiquitin protease in HepG2 and Hep3B cells
Source: PLoS One. 2020 Jun 18;15(6):e0234708. doi: 10.1371/journal.pone.0234708 (PMC7302471; doi:10.1371/journal.pone.0234708)

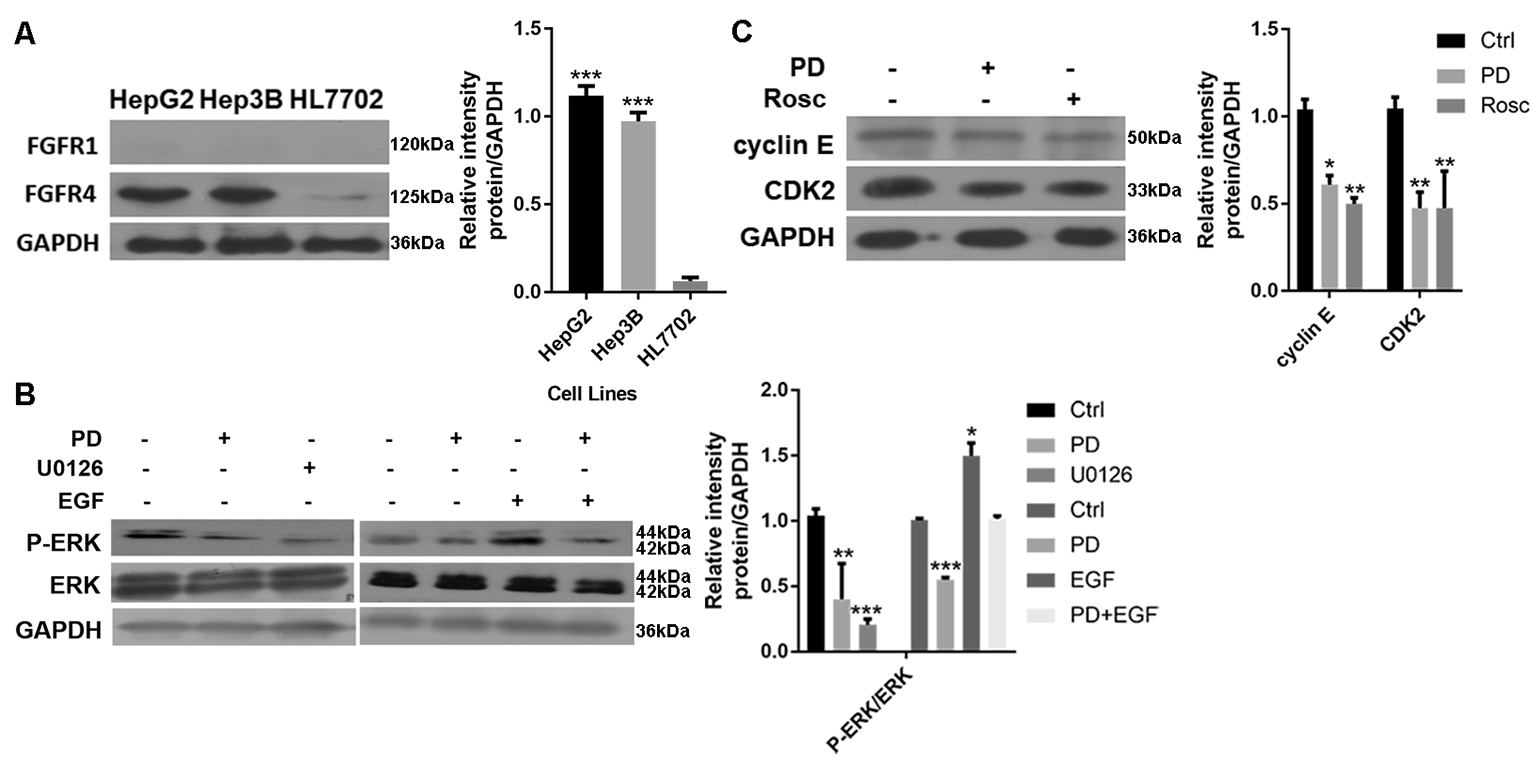

Supplement: S1 Fig — (A) Expression of FGFR1 and FGFR4 in HepG2, Hep3B and HL7702 were determined by Western blot analysis with specific antibodies (n = 3). (B) HepG2 cells were treated with U0126 (2 μM, 4 h), EGF (20 ng/mL, 2 h) or PD (2 μM, 24 h), and ERK phosphorylation was measured by Western blot (n = 3). (C) HepG2 cells were treated with Rosc (4 μM) or PD (2 μM) for 24 h, and cyclin E and CDK2 protein levels were measured by Western blot (n = 3). *P < 0.05, **P < 0.01, ***P < 0.001. Ctrl: control, PD: PD173074, Rosc: roscovitine. (TIF) [file pone.0234708.s001.tif]

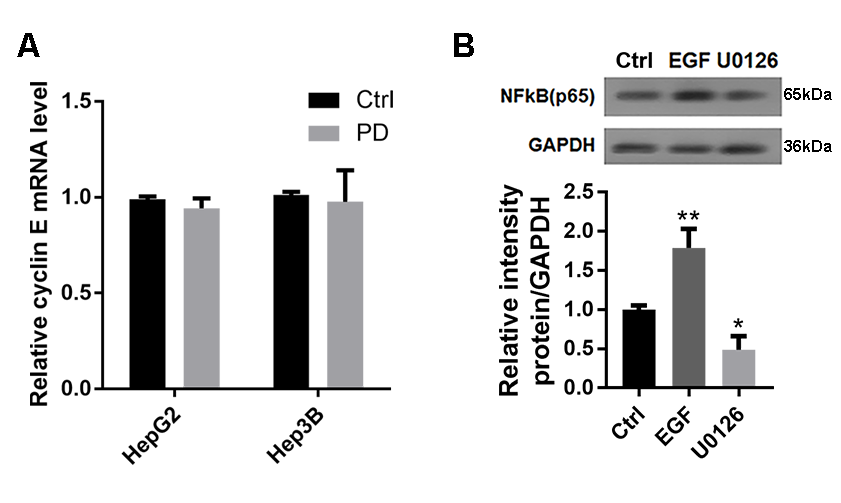

Supplement: S2 Fig — (A) HepG2 and Hep3B cells were treated with PD (2 μM) for 24 h, and RT-qPCR was performed to analyze cyclin E mRNA level. Data were normalized by GAPDH level (n = 5). (B) Western blot analysis of NF-κB (p65) levels in ctrl, EGF- or U0126-treated cells after 2 h and 4 h of treatment, respectively. *P < 0.05, **P < 0.01. PD: PD173074, Ctrl: control, NC: Negative Control. (TIF) [file pone.0234708.s002.tif]
