# Supplementary material for: PD173074 blocks G1/S transition via CUL3-mediated ubiquitin protease in HepG2 and Hep3B cells
Source: PLoS One. 2020 Jun 18;15(6):e0234708. doi: 10.1371/journal.pone.0234708 (PMC7302471; doi:10.1371/journal.pone.0234708)
Supplement: S1 Raw images — (PDF) [file pone.0234708.s003.pdf]

PD173074 blocks G1/S transition via CUL3-mediated ubiquitin protease in HepG2 and Hep3B cells

Chuchu Qiao[1], Hongyan Qian[2], Jue Wang[1], Tingting Zhao[1], Pengyu Ma[1], Sicen Wang[1], Tao Zhang[1]**, Xinshe Liu[2]*

[1] School of Pharmacy, Xi'an Jiaotong University Health Science Center, Xi'an 710061, China.

[2] School of Forensic Science and Medicine, Xi'an Jiaotong University Health Science Center, Xi'an 710061, China.

*Corresponding author. Fax: +86-29-82655117; Tel.: +86-29-82655475; E-mail address: lxins@xjtu.edu.cn (X. Liu)

**Corresponding author. Fax: +86-29-82655451; Tel.: +86-29-82656788; E-mail address: taozhang@xjtu.edu.cn (T. Zhang)

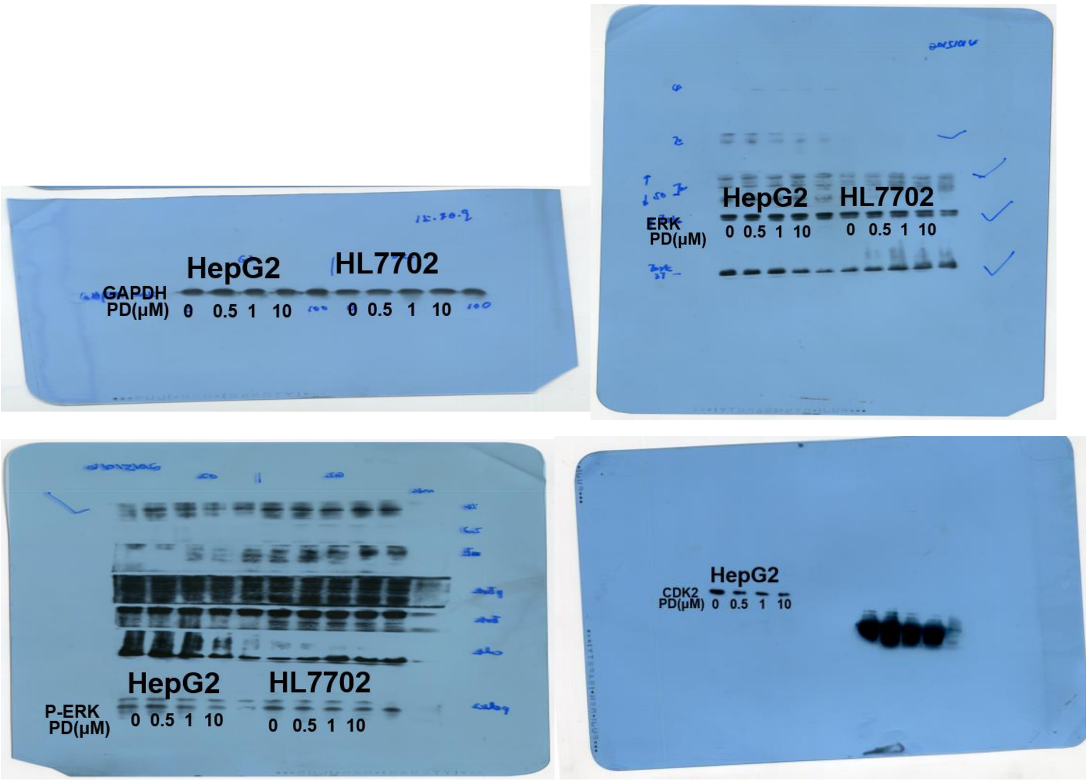

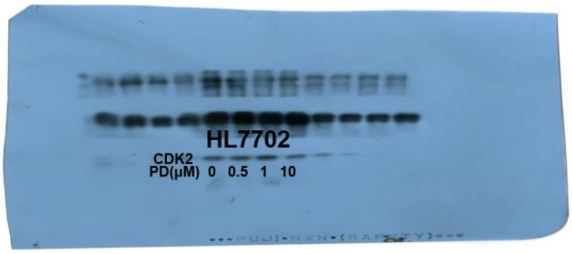
HL7702
CDK2
PD(μM) 0 0.5 1 10

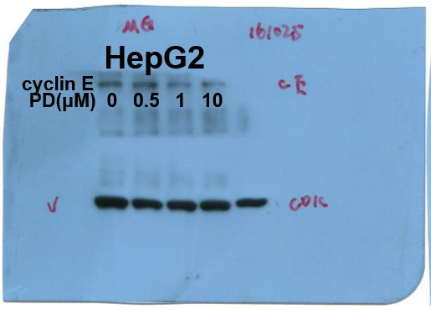
HepG2
cyclin E
PD(μM) 0 0.5 1 10

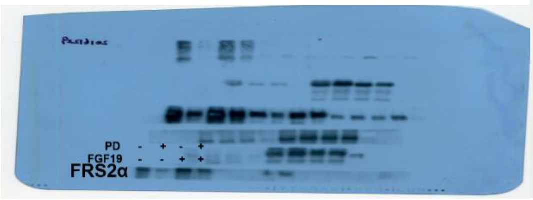
PD - + - +
FGF19 - - + +
FRS2α

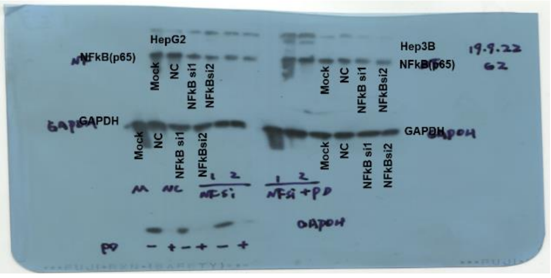
HepG2
NFkB(p65)
Mock
NC
NFkB si1
NFkBsi2
GAPDH
Hep3B
NFkB(p65)
GAPDH

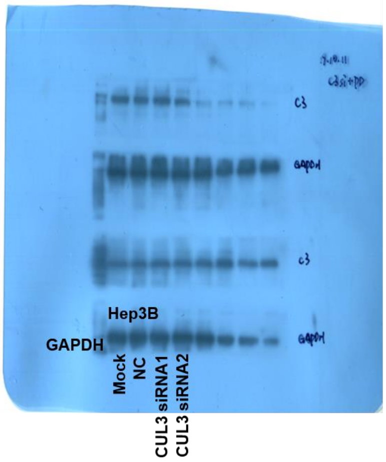
Hep3B
GAPDH
Mock
NC
CUL3 siRNA1
CUL3 siRNA2

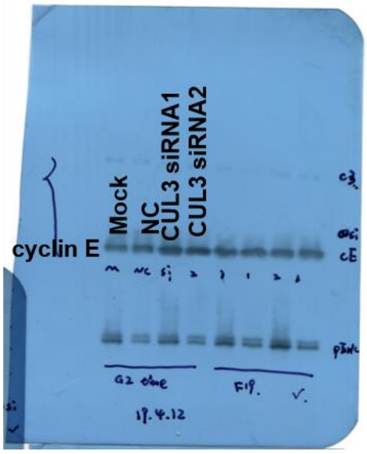
Mock
NC
CUL3 siRNA1
CUL3 siRNA2
cyclin E

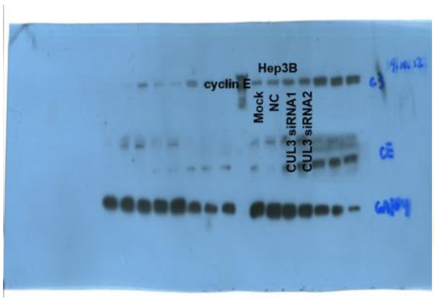
Hep3B
cyclin E
Mock
NC
CUL3 siRNA1
CUL3 siRNA2

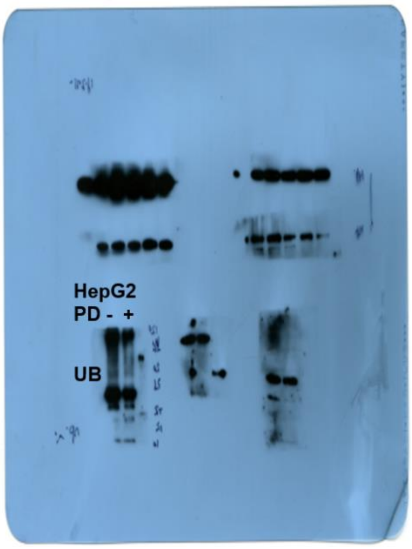
HepG2
PD - +
UB

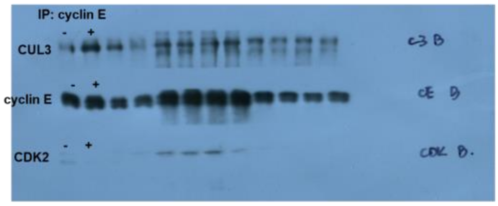
IP: cyclin E
CUL3
cyclin E
CDK2

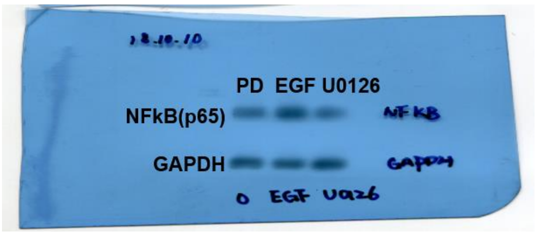
PD EGF U0126
NFkB(p65)
GAPDH
