# Supplementary material for: PD173074 blocks G1/S transition via CUL3-mediated ubiquitin protease in HepG2 and Hep3B cells
Source: PLoS One. 2020 Jun 18;15(6):e0234708. doi: 10.1371/journal.pone.0234708 (PMC7302471; doi:10.1371/journal.pone.0234708)
Supplement: Attachment — Submitted filename: Response to Reviewers.docx [file pone.0234708.s005.docx]

Dear reviewers,

Thank you for your comments concerning our manuscript entitled “PD173074 blocks G1/S transition via CUL3-mediated ubiquitin protease pathway in HepG2 and Hep3B cells”. These comments are very valuable and helpful for improving our manuscript. We have carefully revised the figures and all responds to all comments are as follows:

PONE-D-20-03351R1
PD173074 blocks G1/S transition via CUL3-mediated ubiquitin protease in HepG2 and Hep3B cells
PLOS ONE

Minor comments:

**Question**. Please add scale bars into each figure panel and size markers to each western blot. I also strongly encourage you to upload your original data to an appropriate figure/data repository such as Mendeley Data or Dryad, as access to original data can increase reader confidence in the findings.

**Answer**: Thanks for your suggestion, we have added scale bars into each figure panel (Fig. 1C) and size markers to each western blot (Fig 2, Fig 3, Fig 4, S1 Fig and S2 Fig). We also uploaded our original data to Mendeley Data (http://dx.doi.org/[10.17632/pjryf3kt5d.1](http://dx.doi.org/10.17632/pjryf3kt5d.1)).

Your sincerely

Chuchu Qiao
